# MAM-2201 acute administration impairs motor, sensorimotor, prepulse inhibition, and memory functions in mice: a comparison with its analogue AM-2201

**DOI:** 10.1007/s00213-023-06378-8

**Published:** 2023-05-26

**Authors:** Giorgia Corli, Micaela Tirri, Sabrine Bilel, Raffaella Arfè, Teresa Coccini, Elisa Roda, Beatrice Marchetti, Fabrizio Vincenzi, Giorgio Zauli, Pier Andrea Borea, Carlo Alessandro Locatelli, Katia Varani, Matteo Marti

**Affiliations:** 1 Department of Translational Medicine, Section of Legal Medicine, LTTA Center and University Center of Gender Medicine, University of Ferrara, Via Fossato Di Mortara 17-19, 44121 Ferrara, Italy; 2 Laboratory of Clinical and Experimental Toxicology, and Poison Control Centre and National Toxicology Information Centre, Toxicology Unit, Istituti Clinici Scientifici Maugeri IRCCS, Via Maugeri 10, 27100 Pavia, Italy; 3 Department of Translational Medicine, University of Ferrara, Ferrara, Italy; 4 Research Department, King Khaled Eye Specialistic Hospital, Riyadh, Saudi Arabia; 5 University of Ferrara, Ferrara, Italy; 6 Department of Anti-Drug Policies, Collaborative Center for the Italian National Early Warning System, Presidency of the Council of Ministers, Ferrara, Italy

**Keywords:** AM-2201, MAM-2201, CB_1_ receptor, Sensorimotor responses, Synthetic cannabinoids, Prepulse inhibition, Public health, Occupational risk prevention

## Abstract

**Rationale:**

1-[(5-fluoropentyl)-1*H*-indol-3-yl](4-methyl-1-naphthalenyl) methanone (MAM-2201) is a potent synthetic cannabinoid receptor agonist illegally marketed in “spice” products and as “synthacaine” for its psychoactive effects. It is a naphthoyl-indole derivative which differs from its analogue 1-[(5-Fluoropentyl)-1*H*-indol-3-yl](1-naphthylenyl) methanone (AM-2201) by the presence of a methyl substituent on carbon 4 (C-4) of the naphthoyl moiety. Multiple cases of intoxication and impaired driving have been linked to AM-2201 and MAM-2201 consumption.

**Objectives:**

This study aims to investigate the in vitro (murine and human cannabinoid receptors) and in vivo (CD-1 male mice) pharmacodynamic activity of MAM-2201 and compare its effects with those induced by its desmethylated analogue, AM-2201.

**Results:**

In vitro competition binding studies confirmed that MAM-2201 and AM-2201 possess nanomolar affinity for both CD-1 murine and human CB_1_ and CB_2_ receptors, with preference for the CB_1_ receptor. In agreement with the in vitro binding data, in vivo studies showed that MAM-2201 induces visual, acoustic, and tactile impairments that were fully prevented by pretreatment with CB_1_ receptor antagonist/partial agonist AM-251, indicating a CB_1_ receptor mediated mechanism of action. Administration of MAM-2201 also altered locomotor activity and PPI responses of mice, pointing out its detrimental effect on motor and sensory gating functions and confirming its potential use liability. MAM-2201 and AM-2201 also caused deficits in short- and long-term working memory.

**Conclusion:**

These findings point to the potential public health burden that these synthetic cannabinoids may pose, with particular emphasis on impaired driving and workplace performance.

## Introduction

### Prevalence of synthetic cannabinoids

**Fig. 1:**
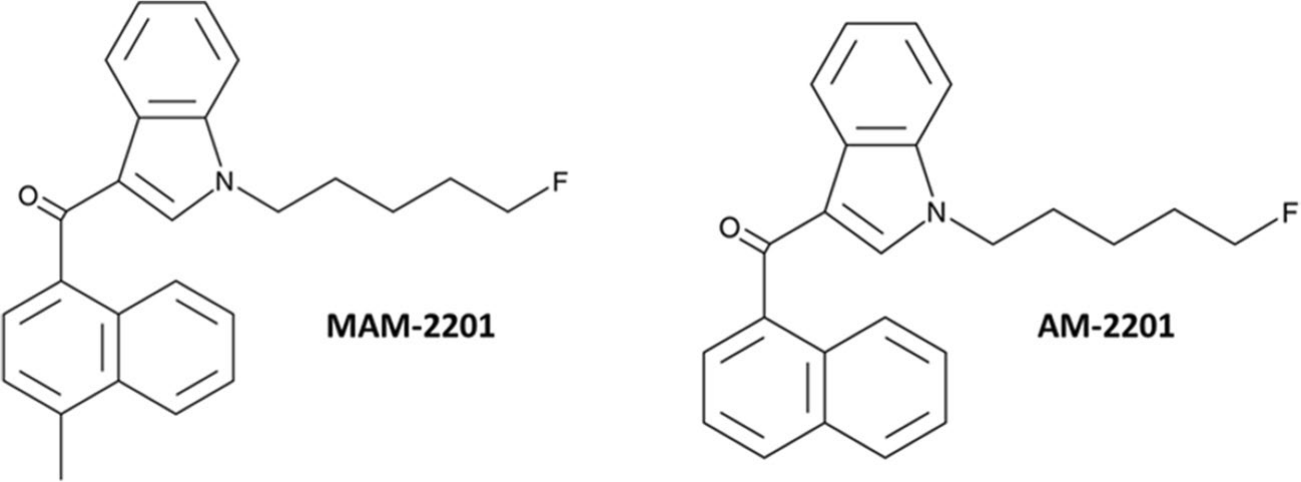
Chemical structures of MAM-2201 (1-(5-fluoropentyl)- 1H-indol-3-yl](4-methyl-1-naphthalenyl)-methanone) and AM-2201 (1-(5-fluoropentyl)-3-(1-naphthoyl)indole; Cayman chemicals)

As reported by European authorities, synthetic cannabinoids (SCs) have been among the most frequently used and seized class of novel psychoactive substances (NPS) in the last few years (EMCDDA 2022a, b). SCs mimic the effect of the main psychoactive component of *Cannabis* (i.e., THC) but are linked to more serious adverse effects due to their greater agonist action on cannabinoid receptors (CB_1_ and CB_2_; Tai and Fantegrossi 2017; Luethi and Liechtie 2020). These substances are mainly contained in herbal mixtures usually sold online as “research chemical,” “herbal incense,” or “legal high,” under different brand names such as “K2” or “spice” (Marusich et al. 2018). Since the first appearance in the market, many new compounds have been synthetized through chemical modifications of the main structures, which allows the analogs to avoid legal restrictions and law enforcement efforts aimed to discourage manufacture, distribution, and use. Thus, continuous chemical evolution has led to different generations of SCs appearing on the market over time. Among these, the well-known naphtoyl-indole JWH-018 is considered as the parent molecule of the first generation SCs. Notably, several substituted JWH-018 analogs, such as AM-2201 (1-[(5-fluoropentyl)-1*H*-indol-3-yl](1-naphthylenyl) methanone), have appeared on the illicit drug market in the last decade. Together with the halogenated derivative of JWH-018, AM-2201, its methylated analogue (1-[(5-fluoropentyl)- 1*H*-indol-3-yl](4-methyl-1-naphthalenyl) methanone), also known as MAM-2201, has been widely detected in spice-like herbal smoking mixtures (Fig. 1). These compounds have been mainly seized in Europe and USA. In particular, MAM-2201 has been seized in Bulgaria, Australia, and Japan (Shanks et al. 2013; Marusish et al. 2018; Andreeva-Gateva et al. 2015), as well as in Finland in the crystalline powder-product labeled as synthacaine, which is usually sold online as “legal cocaine” (Jang et al. 2014; Lonati et al. 2014).

### Pharmacology

Like other SCs, MAM-2201 acts as a potent full agonist of CB_1_ receptors (Ki = 2.07 ± 0.82 nM), that are mainly positioned at neuronal presynaptic terminals, modifying central and peripheral processes, but also exerts actions on CB_2_ receptors (Ki = 0.582 ± 0.123 nM) receptor, which are mostly situated on peripheral immune cells (Howlett et al. 2002; Hess et al. 2015). Similarly, AM-2201 retains nanomolar affinity for both CB_1_ (K_i_ = 1 nM) and CB_2_ (K_i_ = 2.6 nM) receptors (Hess et al. 2015). In line with structural similarities between MAM-2201 and AM-2201, recreational users on online forums have described comparable effects following the abuse of these substances (Bluelight n.d; Eve-rave n.d). Multiple cases of intoxication describing people aged 20–30 years involved in vehicle accidents have been linked to AM-2201 and MAM-2201 consumption. Moreover, psychotic symptoms such as confusion, disturbance of motor skills, blunt mood, and enlarged pupils have been reported in drivers affected by the use of AM-2201 (Yeakel and Logan 2013; Musshoff et al. 2014; Elian and Hackett 2014) and individuals manifesting seizures after smoking spice product containing this substance have also been reported (McQuade et al. 2013). Likewise, data in the literature about MAM-2201-related intoxications cases show that this compound can induce symptoms such as retarded movement sequences, apathy, nervousness, inertness, delayed reaction of pupils to light, as well as excitatory symptoms when taken alone or with other substances (synthetic cathinones; Lonati et al. 2014; Musshoff et al. 2014; Kaneko 2017). It is worth noting that MAM-2201 has been also reported to provoke long-lasting effects, including panic attacks and vomiting at higher doses (Derungs et al. 2013). Considering these adverse consequences on human health, public concern is not limited to SCs used by adolescents, young and adults (i.e., general population), but also to NPS abuse among workers in occupational settings (Dobaja et al. 2017; Tapp et al. 2014; EMCDDA 2022a, b).

### Metabolism and pharmacodynamics

In addition, pharmaco-toxicological effects induced by compounds such as AM-2201 and MAM-2201 may be also influenced by their metabolic profile (Tai and Fantegrossi 2017; Jang et al. 2014). Specifically, in vitro studies investigating pharmacokinetics of MAM-2201 have reported mono- and di-hydroxy side chain metabolites ofMAM-2201, such as N-(4-hydroxypentyl)-MAM-2201, N-(5-hydroxypentyl)-MAM-2201, and MAM-2201 N-pentanoic acid as major metabolites (Kong et al. 2017a). In addition, Kim and colleagues have identified 13 novel metabolites, including one phase I metabolite and 12 phase II metabolites (6 glucoronides, hydroxy-MAM-2201 sulfate and 5 GSH conjugates; Kim et al. 2018). Further studies investigating enzymes involved in MAM-2201 metabolic pathways have shown that it is substrate of phase I metabolism enzymes CYP3A4, CYP1A2, CYP2B6, CYP2C8, and CYP2C9, while enzyme UGT1A3 has been shown to play a key role in phase II metabolism (Kong et al. 2017b). This is particularly relevant to better evaluate potential drug interaction between SCs and other substances. Notably, both AM-2201 and MAM-2201 have been also shown to be competitive inhibitors of CYP2C8, CYP2C9, CYP3A4, and UGT1A3 mediated metabolism reactions involving several drugs (Kim et al. 2017; Kong et al. 2017b). In vitro electrophysiological studies carried out on cerebellum of rodents have, moreover, showed cytotoxicity potential of both MAM-2201 and AM-2201 on primary neuronal cells of the forebrain that was reversed by the cannabinoid receptor antagonist (AM-251), emphasizing that even this detrimental effect of SCs is related to their action on CB_1_ receptors (Tomiyama and Funada 2014). In line with these findings, Coccini and colleagues have recently revealed cytotoxic effects provoked by MAM-2201 on human cell-based models of neurons and astrocytes (Coccini et al. 2021). Noteworthy, previous in vivo studies have investigated AM-2201-induced acute effects in rodents. Specifically, it has been pointed out that this compound alters body temperature and blood pressure in rats (Schindler et al. 2017), as well as induces seizures in mice via CB_1_ receptors-mediated mechanisms that lead to enhanced glutamatergic transmission in the hippocampus (Funada and Takebayashi-Ohsawar 2018). Moreover, disruption of sensorimotor responses has been recently shown after administration of AM-2201 and other halogenated derivatives of JWH-018 (Bilel et al. 2020). Nevertheless, scarce information is available at present about in vivo effects induced by its methylated analogue MAM-2201. Therefore, this study aims to characterize the in vitro and in vivo pharmaco-dynamic profile of MAM-2201. Both compounds were tested through in vitro cyclic AMP and binding competition experiments to evaluate their potency and affinity for murine and human CB_1_ and CB_2_ receptors. In addition, effects induced by acute administration of this compound on sensorimotor (visual, acoustic, and tactile) responses have been evaluated and compared with those induced by AM-2201. An antagonistic study was also performed to investigate whether MAM-2201 affects sensorimotor functions through a CB_1_-mediated action. Moreover, its effects on motor (open field) and sensory gating (PPI) functions have been investigated. Finally, the effects of MAM-2201 and AM-2201 on recognition and memory function using a novel object recognition assay (NOR) have been assessed.

## Materials and methods

### Animals

Male ICR (CD-1®) mice, 3–4 months old, weighing 25–30 gr (ENVIGO Harlan Italy, Italy; bred inside the Laboratory for Preclinical Research (LARP) of the University of Ferrara, Italy), were group-housed (5 mice per cage; floor area/animal: 80 cm^2^; minimum enclosure height: 12 cm) on a 12:12-h light–dark cycle (light on at 6:30 AM), temperature of 20–22 °C, humidity of 45–55% and were provided ad libitum access to food (Diet 4RF25 GLP; Mucedola, Settimo Milanese, Milan, Italy) and water. The experimental protocols were in accordance with the European Communities Council Directive of September 2010 (2010/63/EU) a revision of the Directive 86/609/EEC were approved by the Ethics Committee of the University of Ferrara and by Italian Ministry of Health (auth. num. 335/2016-PR). Moreover, adequate measures were taken to reduce the number of animals used and their pain and discomfort according to the ARRIVE guidelines.

According to the last European School Survey Project on Alcohol and Other Drugs (ESPAD), the lifetime prevalence of the use of SCs has been higher among male young adults than female in the last years (ESPAD 2019). In line with these epidemiological data, a higher prevalence of cases of DUID involving SCs has been recently estimated for male drivers in respect to female by Pelletti and colleagues (Pelletti et al. 2022). Thus, male mice were used in this study. However, it has been pointed out that women displayed stronger cognitive and psychomotor impairment when tested in Driving Under the Influence (DUI) of high dosages of cannabis (Spindle et al. 2021), suggesting the relevance of sex-based differences that should be investigated. Therefore, further research must be conducted to clarify this point.

### Drug preparation and dose selection

MAM-2201 and AM-2201 were purchased from LGC Standards (LGC Standards, Milan, Italy) and AM-251 was purchased from Tocris (Tocris, Bristol, UK). Drugs for in vivo testing were initially dissolved in absolute ethanol (final concentration: 5%) and Tween 80 (final concentration: 2%) and brought to the final volume with saline (0.9% NaCl). The solution made with ethanol, Tween 80, and saline was also used as the vehicle. The CB_1_ receptor-preferring antagonist/inverse agonist AM 251 (6 mg/kg) was administered 20 min before MAM-2201 injection. Drugs were administered by intraperitoneal route at a volume of 4 ul/g. Based on previous studies (Ossato et al. 2015; Bilel et al. 2020), the range of MAM-2201 doses (0.01–6 mg/kg i.p.) was chosen to compare the effects of AM-2201 on sensorimotor function with those induced by the MAM-2201.

### In vitro* studies*

#### Mouse brain and spleen membrane preparation

To evaluate the affinity of synthetic cannabinoids for murine CB_1_ and CB_2_ receptors, membranes from mouse brain and spleen were used, respectively. Following excision from mice, tissues were suspended in 50 mM Tris HCl, pH 7.4 at 4 °C. The mouse brain and spleen tissues were homogenized with a Polytron and subsequently centrifuged for 10 min at 2000 × g. The resulting supernatants were filtered, centrifuged for 20 min at 40,000 × g and the pellets were used for competition binding experiments (Vincenzi et al. 2013).

#### Cell culture and membrane preparation

CHO cells transfected with human CB_1_ or CB_2_ receptors (Perkin Elmer Life and Analytical Sciences, USA) were grown adherently and maintained in Ham’s F12 containing 10% fetal bovine serum, penicillin (100 U/ml), streptomycin (100 µg/ml), and Geneticin (G418, 0.4 mg/ml) at 37 °C in 5% CO_2_/95% air. To obtain membranes, cells were washed with PBS and scraped off with ice-cold hypotonic buffer (5 mM Tris HCl, 2 mM EDTA, pH 7.4). The cell suspension was homogenized with a Polytron and then centrifuged for 30 min at 40,000 × g. The membrane pellet was suspended in 50 mM Tris HCl buffer (pH 7.4) containing 2.5 mM EDTA, 5 mM MgCl_2_, 0.5% BSA for CB_1_ receptors or in 50 mM Tris HCl (pH 7.4), 1 mM EDTA, 5 mM MgCl_2_, 0.5% BSA for CB_2_ receptors (Vincenzi et al. 2013).

#### ***[***^***3***^***H] CP-55,940 competition binding assays***

Competition binding experiments were carried out incubating 0.5 nM [^3^H]-CP-55,940 (Perkin Elmer Life and Analytical Sciences, USA) and different concentrations of the tested compounds for 90 or 60 min at 30 °C for CB_1_ or CB_2_ receptors, respectively. For human cannabinoid receptors, membranes obtained from CHO cells transfected with human CB_1_ or CB_2_ receptors (2 µg protein/100 µl) were used. Competition binding experiments at murine cannabinoid receptors were performed with mouse brain membranes (40 µg protein/100 µl) or with mouse spleen membranes (80 µg protein/100 µl) for CB_1_ receptors or CB_2_ receptors, respectively. Non-specific binding was determined in the presence of 1 mM WIN 55,212–2 (Vincenzi et al. 2013). Bound and free radioactivity were separated by filtering the assay mixture through Whatman GF/C glass fiber filters using a Brandel cell harvester (Brandel Instruments, Unterföhring, Germany). The filter-bound radioactivity was counted using a Packard Tri Carb 2810 TR scintillation counter (Perkin Elmer Life and Analytical Sciences, USA).

#### Cyclic AMP assays

CHO cells transfected with human CB1 or CB2 receptors were washed with PBS, detached with trypsin, and centrifuged for 10 min at 200 × g. The pellet containing 1 × 10^6^ cells/assay was suspended in 0.5 ml of buffer containing150 mM NaCl, 2.7 mM KCl, 0.37 mM NaH2PO4, 1 mM MgSO4, 1 mM CaCl2, 5 mM HEPES, 10 mM MgCl2, and 5 mM glucose, at pH 7.4 and 37 °C. Cells were pre-incubated with 0.5 mM of the phosphodiesterase inhibitor 4-(3-butoxy-4-methoxybenzyl)-2-imidazolidinone (Ro 20–1724;) for 10 min in a shaking bath at 37 °C. The potency of the examined compounds to inhibit adenylate cyclase activity was determined in the presence of forskolin 1-µM stimulation. The reaction was terminated by the addition of cold 6% trichloroacetic acid (TCA) and the final aqueous solution was tested for cyclic AMP levels by a competition protein binding assay (AlphaScreen cAMP Detection Kit, Cat. Number 6760635D, Perkin Elmer Life and Analytical Sciences, USA) following the manufacturer’s instructions; Vincenzi et al. 2013).

#### Data analysis

The protein concentration was determined according to a Bio-Rad method (500–0006; Bio-Rad Laboratories, Hercules, CA, USA) with bovine serum albumin as a reference standard. Inhibitory binding constants, Ki, were calculated from the IC_50_ values according to the Cheng and Prusoff equation: Ki = IC_50_/(1 + [C*]/K_D_*), where [C*] is the concentration of the radioligand and K_D_* its dissociation constant. Functional experiments were analyzed by non-linear regression analysis using the equation for a sigmoid concentration–response curve (GraphPad Prism, USA). All the data are expressed as the mean ± SEM of three independent experiments.

### In vivo* studies*

#### Behavioral tests

For the overall study 234 mice were used. In sensorimotor (visual object, visual placing, acoustic, and overall tactile responses) tests for MAM-2201, experiments for each treatment dose (vehicle or 5 different MAM-2201 doses, 0.01, 0.1, 1, 3, and 6 mg/kg) used 8 mice (total mice used: 48) and for MAM-2201 with AM-251 pretreatment, experiments for each dose regimen (vehicle + AM-251 or MAM-2201 + AM-251, 6 mg/kg) used 16 mice. In the analysis of spontaneous locomotion (open field test) for MAM-2201, experiments for each treatment regimen (vehicle or 4 different MAM-2201 doses, 0.01, 0.1, 1, and 6 mg/kg) used 10 mice (total mice used: 50). In the PPI test for MAM-2201, experiments for each treatment (vehicle or 3 different MAM-2201 doses, 0.01, 0.1, and 1 mg/kg) used 10 mice (total mice used: 40), while for MAM-2201 experiments in the Novel Object Recognition (NOR) test for each treatment regimen (vehicle or 3 different MAM-2201 and vehicle or 3 different AM-2201 doses, 0.01, 0.1, and 1 mg/kg) used 10 mice (total mice used: 80). In the present study the effect of MAM-2201 on sensorimotor responses was investigated using a battery of behavioral tests widely used in studies of “safety-pharmacology,” that we routinely adopted in our laboratory, for the preclinical characterization of new molecules in rodents (Ossato et al. 2015; Vigolo et al. 2015; Bilel et al. 2020). The voluntary and involuntary motor responses of the animal to different visual, acoustic, and tactile stimuli were evaluated according to the procedure described in our previous studies (Ossato et al. 2015; Bilel et al. 2020). To reduce the number of animals used, mice were evaluated in functional observational tests carried out in a consecutive manner according to the following time scheme: observation of visual object responses (frontal and lateral view), acoustic response, tactile response (vibrissae, corneal and pinnae reflexes), and visual placing response. Sensorimotor tests were measured at 10, 30, 60, 120, 180, 240, and 300 min after injections for the evaluation of the visual object, acoustic and the tactile response, and at 15, 45, 75, 135, 195, 255, and 315 min after injections for the evaluation of visual placing response. Behavioral tests were conducted in a thermostated (temperature: 20–22 °C, humidity: 45–55%) and light (150 lx) controlled room with a background noise of 40 ± 4 dB. The apparatus for the visual object, acoustic and tactile sensorimotor tests consisted of an experimental chamber (350 × 350 × 350 mm) with black methacrylate walls and a transparent front door. During the week before the experiment, each mouse was placed in the box and handled (once a day) on every other day, i.e., 3 times, to get used to both the environment and the experimenter. To avoid olfactory cues, cages were carefully cleaned with a dilute (5%) ethanol solution and rinsed with water. All experiments were performed between 8:30 AM and 2:00 PM and conducted by trained observers working in pairs who were blinded to the treatment being tested (Ossato et al. 2015). The behavior of mice was videotaped by a camera (B/W USB Camera day & night with varifocal lens; Ugo Basile, Italy) placed at the top or on one side of the box and analyzed off-line by a different trained operator. Spontaneous locomotion studies were conducted in a separate set of animals through ANYMAZE test. Sensory gating was evaluated by analysis of the pre-pulse inhibition (PPI) of the acoustic startle reflex as previously described (Bilel et al. 2020).

##### Evaluation of the visual response

Visual response was verified by two behavioral tests which evaluated the ability of the animal to capture visual information when the animal is either stationary (the visual object response) or moving (the visual placing response).

*Visual object response* test was used to evaluate the ability of the mouse to see an object approaching from the front (frontal view) or the side (lateral view) that typically induces the animal to shift or turn the head, bring the forelimbs in the position of “defense” or retreat from it. For the frontal visual response, a white horizontal bar was moved frontally to the mouse head and the maneuver was repeated 3 times. For the lateral visual response, a small dentist’s mirror was moved into the mouse’s field of view in a horizontal arc, until the stimulus was between the mouse’s eyes. The procedure was conducted bilaterally (Ossato et al. 2015; Bilel et al. 2020) and was repeated 3 times. The score assigned was 1 if there was a reflection in the mouse movement or 0 if it was not present. The total value was calculated by adding the scores obtained in the frontal with those obtained in the lateral visual object response test (overall score: 9).

*Visual Placing response* test is performed using a tail suspension modified apparatus able to bring the suspended mouse down towards the floor at a constant speed of 10 cm/s (Ossato et al. 2015; Bilel et al. 2020). Briefly, CD-1 mice were suspended 20 cm above the floor by an adhesive tape placed approximately 1 cm from the tip of the tail. The downward movement of the mouse was videotaped by a camera (B/W USB Camera day & night with varifocal lens; Ugo Basile, Italy) placed at the base of the tail suspension apparatus. Movies were analyzed off-line by a trained operator who was unaware of the drug treatments performed. The frame by frame analysis allows evaluating the beginning of the reaction of the mouse while it was approaching the floor. The first movement of the mouse when it perceives the floor is the extension of the front legs. When the mouse started this reaction, an electronic ruler evaluated the perpendicular distance in millimeters between the eyes of the mouse to the floor. Untreated control mice typically perceive the floor and prepare to contact at a distance of about 23.6 ± 4.8 mm.

##### Evaluation of acoustic response

Acoustic response measures the reflex of the mouse in response to an acoustic stimulus produced behind the animal (Ossato et al. 2015; Bilel et al. 2020). In particular, the four acoustic stimuli of different intensity and frequency were tested: a snap of the fingers (four snaps repeated in 1.5 s), a sharp click (produced by a metal instrument; four clicks repeated in 1.5 s), an acute sound (produced by an audiometer; frequency: 5.0–5.1 kHz), and a severe sound (produced by an audiometer; frequency: 125–150 Hz). Each test was repeated 3 times. The score assigned was 1 if there was a response or 0 if it was not present, for a total score of 3 for each sound. The acoustic total score was calculated by adding the scores obtained in the four tests (overall score: 12). The background noise (about 40 ± 4 dB) and the sound from the instruments were measured with a digital sound level meter.

##### Evaluation of tactile response

Tactile response in the mouse was verified through vibrissae, corneal, and pinnae reflexes (Ossato et al. 2015; Bilel et al. 2020). Data is expressed as the sum of the three above-mentioned parameters. *Vibrissae reflex* was evaluated by touching vibrissae (right and left) with a thin hypodermic needle once per side giving a value of 1 if there was a reflex (turning of the head to the side of touch or vibrissae movement) or 0 if not present (overall score: 2). *Corneal reflex* was assessed by gently touching the cornea of the mouse with a thin gavage plastic needle and evaluating the response: the score assigned was 1 if the mouse moved only the head, 2 if it only closed the eyelid, 3 if it closed the lid and moved the head. The procedure was conducted bilaterally (overall score: 6). *Pinna reflex* was assessed by touching pavilions (left and right) with a thin hypodermic needle: first the interior pavilions and then the external. This test was repeated twice for each side giving a score of 1 if a reflex was present and 0 if it was not present (overall score: 4).

##### Spontaneous locomotor activity

Spontaneous locomotor activity was measured by using the ANY-maze video tracking system (Ugo Basile, application version 4.99 g Beta). As previously reported, the mouse was placed in a square plastic cage (60 × 60 cm) located in a sound- and light-attenuated room and motor activity was monitored for 240 min (Ossato et al. 2015; Bilel et al. 2020). The four mice were monitored at the same time in each experiment. Measured parameters were: distance traveled (m), immobility time (s; the animal is considered immobile when 95% of his image remains in the same place for at least 2 s) and maximum speed (m/s). Variations of the distance traveled and the time of immobility were repeatedly analyzed setting up test intervals of 15 min for a maximum time of observation of 240 min. To avoid olfactory cues, cages were carefully cleaned with a dilute (5%) ethanol solution and rinsed with water between animal trials. All experiments were performed between 9:00 AM and 1:00 PM.

##### Acoustic Startle and Pre-Pulse inhibition (PPI) test

As previously reported (Ossato et al. 2015; Bilel et al. 2020), mice were tested for acoustic startle reactivity and pre-pulse inhibition in startle chambers (Ugo Basile apparatus, Milan, Italy) consisting of a sound-attenuated, lighted, and ventilated enclosure holding a transparent non-restrictive Perspex® animal cage (90 × 45 × 50 mm). A loudspeaker mounted laterally within the animal holder produced all acoustic stimuli. The wave amplitude evoked by the movement of the animals’ startle response were detected by a loadcell. At the onset of the startling stimulus, 300-ms readings were recorded and the wave amplitude was measured.

Acoustic startle test sessions consisted of startle trials (pulse-alone) and prepulse trials (prepulse + pulse). The pulse-alone trial consisted of a 40-ms 120-dB pulse. Prepulse + pulse trials sequence consisted of a 20-ms acoustic prepulse, 80-ms delay, and then a 40-ms 120-dB startle pulse (100-ms onset–onset). There was an average of 15 s (randomly ranging from 9 to 21 s) between the trials. Each startle session began with a 10-min acclimation period with a 65-dB broadband white noise that was present continuously throughout the session. The test session contained 40 trials composed by pulse-alone and prepulse + pulse trials (with three different prepulses of 68-dB, 75-dB, and 85-dB) presented in a pseudo-randomized order. MAM-2201 was administered intraperitoneally, and animals were placed in the startle chambers 5 min after treatments. Mice were left in the chambers for 10 min (acclimation period) and the entire PPI test lasted 20 min. The amount of PPI was expressed as the percentage decrease in the amplitude of the startle reactivity caused by the presentation of the prepulse (% PPI).

##### Novel Object Recognition test

The Novel Object Recognition (NOR) test was chosen as it represents a ‘‘pure’’ working memory task, which does not involve the retention of a rule, but it is entirely based on the spontaneous exploratory behavior of rodents towards objects (Ennaceur and Delacour 1988; Ennaceur and Meliani 1992; Scali et al. 1997; Ennaceur 2010).

This test was performed according to the method reported by Ennaceur and Delacour (1988) and Antunes and Biala (2012). The test was conducted in three phases: habituation, familiarization, and choice. Firstly, CD-1 mice (*n* = 10/group) were subjected to a 3-day habituation phase, conducted by placing each animal into the NOR chamber (a square open field 60 cm × 60 cm × 40 cm, dark PVC plastic box) located in a dimly lit (50 lx), sound-attenuated and acclimatized room. Mice were allowed to explore freely for 20 min/day. No objects were placed in the box during the habituation trial. Twenty-four hours (h) after last habituation section, the familiarization trial was conducted by placing the mouse in the field in which two identical objects (A, A) were positioned in the corners of the arena approximately 6 cm from the walls. Mice were placed at the mid-point of the wall opposite to the objects and allowed to explore them for 15 min. Fifteen minutes after the familiarization phase, mice were injected with vehicle or drug (MAM-2201 and AM-2201) and tested in two consecutive choice sections performed 2 h (short-term memory) and 24 h (long-term memory) after the drug administration. During the choice test at 2 h, one of the two familiar objects (A) was replaced with a new one (novel; B), different in shape, dimension, and color. Each mouse was then placed in the apparatus and left free to explore the objects (A and B) for 5 min. In the choice test given at 24 h, the mice explored the open field for 5 min in the presence of one familiar (A) and one novel object (C, different from B). Exploration was defined as the time (s) during which the mouse nose was in contact with the object or directed toward it at a distance ≤ 2 cm. Turning around the object was not considered as exploratory behavior.

All experiments were performed using the ANY-maze video tracking system (Ugo Basile, application version 4.99 g Beta) and subsequently analyzed by an observer blind to the mouse treatment and to which object was the novel one. Exploration time of familiar (A) and novel (B) object was detected. The novel object preference was quantified as the Recognition Index (RI) calculated as: $RI=\frac{\mathrm{novel B}-\mathrm{familiar A}}{\mathrm{novel B}+\mathrm{familiar A}}.$ Using this metric, scores approaching zero reflects no preference (impairment of recognition memory), positive values reflect preference for the novel object (good recognition memory) while negative numbers reflect preference for the familiar (impairment of recognition memory). Moreover, the total exploration time (s) spent by the animal in the choice phase at 2 h (familiar A + novel B) and 24 h (familiar A + novel C) was calculated to investigate the effect of drugs on object exploration.

The objects to be discriminated by mice were 7 sets of novel and familiar objects of different material (plastic, glass, or ceramic), shape (cube, parallelepiped, and cylinder), dimension (height: 3–8 cm; width: 6–8), and color (light yellow, red and blue). The set of objects used in the familiarization phase (two identical A, A objects) was used in the subsequent vehicle/drug conditions at 2 and 24 h. The choice of object for novel or familiar was counterbalanced and the position of each object was also alternated between trials to avoid any misinterpretation of data. The object weight was such that they could not be displaced by mice. To avoid mice olfactory cues, objects and apparatus were carefully cleaned with a dilute (5%) ethanol solution and water between animal trials and also between familiarization and choice phase (executed 2 and 24 h after the familiarization phase). Animals that spent less than 10 s exploring both objects were excluded from the study and replaced by other animals.

#### Statistical analysis

In sensorimotor response, experiments data are expressed in arbitrary units (visual objects response, acoustic response, vibrissae, corneal, and pinnae reflex) and percentage of baseline (visual placing response). Data from spontaneous locomotion studies are expressed in absolute values for the total distance traveled (m), immobility time (s), and maximum speed (m/s). The amount of PPI was calculated as a percentage score for each prepulse + pulse trial type: $\%PPI=100-\left\{\left[\frac{\left(\mathrm{startle response for prepulse}+\mathrm{pulse trial}\right)}{\left(\mathrm{startle response for pulse}-\mathrm{alone trial}\right)}\right]\times 100\right\}.$ Startle magnitude was calculated as the average response to all of the pulse-alone trials. All data are shown as mean ± SEM of 4 independent experimental replications. Statistical analysis of the effects of each compound at different concentrations over time and of those of the cannabinoid antagonist was performed by two-way ANOVA followed by Bonferroni post hoc test for multiple comparisons. Analysis of the total average effect induced by treatments was performed with one-way ANOVA followed by Bonferroni’s post hoc test for multiple comparisons. Statistical analysis was performed using the program Prism software (GraphPad Prism, USA). ED_50_ (dose of agonist to obtain 50% of the overall mean effect) values were calculated by non-linear regression analysis of dose–response data performed using the Prism software (GraphPad Prism, San Diego CA). The calculation of AM-2201 ED_50_ values was based on previous studies results (Bilel et al. 2020). Curves have been compared performing the F test (curves comparison).

## Results

### *Affinity and potency of the examined compounds for CB*_*1*_* and CB*_*2*_* receptors*

Competition binding experiments performed in CHO cell membranes transfected with human CB_1_ or CB_2_ receptors revealed affinity values in the low nanomolar range for both the tested compounds AM-2201 and MAM-2201 (Table 1). Comparable results were obtained evaluating affinity values of the two synthetic cannabinoids in mouse tissues suggesting no species selectivity between murine and human CB receptors. Interestingly, AM-2201 and MAM-2201 displayed a higher affinity for CB_1_ than CB_2_ receptors, with a selectivity index (ratio between the Ki value to human CB_2_ and the Ki value to human CB_1_) of 9.9 and 9.3 for human CB receptors and of 7.4 and 9.0 for murine CB receptors, respectively. For both human and mouse CB receptors, MAM-2201 revealed a slightly higher affinity than AM-2201 (Table 1).

**Table 1:** Binding and functional parameters of MAM-2201 and AM-2201 to human and mouse CB_1_ and CB_2_ receptors

Compound	hCB_1_ CHO membranes^a^ Ki (nM)	hCB_2_ CHO membranes^a^ Ki (nM)	Mouse cortex membranes CB_1_^a^ Ki (nM)	Mouse spleen membranes CB_2_^a^ Ki (nM)	hCB_1_ CHO cells^b^ IC_50_ (nM)	hCB_2_ CHO cells^b^ IC_50_ (nM)
AM-2201	1.66 ± 0.13	16.4 ± 1.5	1.96 ± 0.13	14.6 ± 1.2	2.84 ± 0.21	31.8 ± 2.9
MAM-2201	1.11 ± 0.12	10.3 ± 2.1	1.07 ± 0.23	9.6 ± 1.9	1.26 ± 0.56	22.4 ± 3.8

Data are expressed as mean ± SEM

^a^[^3^H]-CP-55,940 competition binding experiments

^b^Cyclic AMP experiments

Cyclic AMP experiments were performed to evaluate the potency of the examined compounds in CHO cells transfected with human CB_1_ or CB_2_ receptors. Both synthetic cannabinoids were more potent at CB_1_ than CB_2_ receptors (Table 1). In agreement with binding data, MAM-2201 showed a higher potency value in comparison with AM-2201. The tested compounds were able to completely inhibit the forskolin-stimulated cAMP production, thus behaving as full agonists.

### Evaluation of the visual object response

**Fig. 2:**
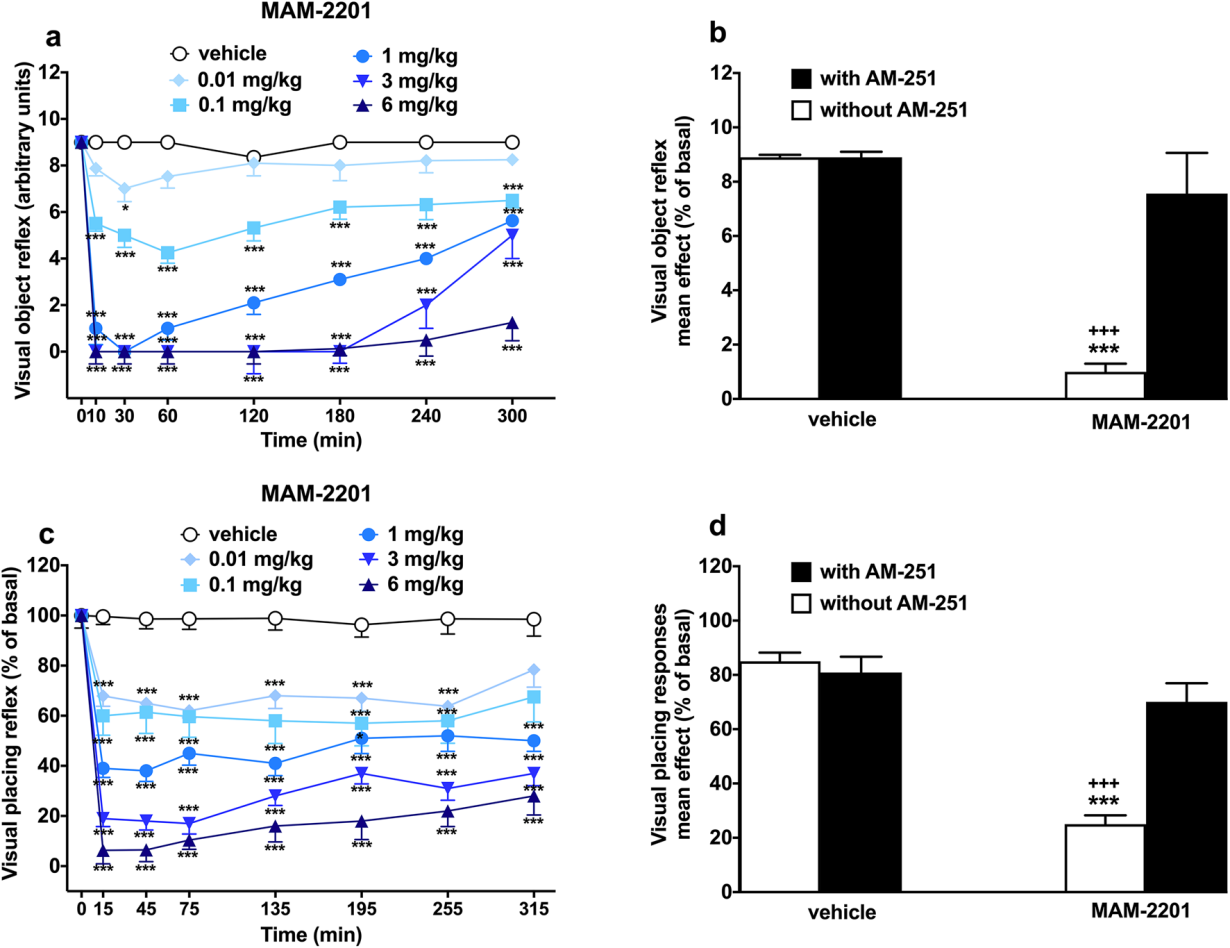
Effect of MAM-2201 (0.01–6 mg/kg i.p.) injections on the visual object (**a**) and visual placing (**c**) response in mice. Data are expressed as arbitrary units (**a**, **b**) or percentage of baseline (**c**, **d**) and represent the mean ± SEM of 8 determinations for each treatment. Interaction of MAM-2201 (6 mg/kg) with the selective CB1 receptor antagonist AM-251 (6 mg/kg, i.p.) is shown in panels **b** and **d**. Statistical analysis was performed by two-way ANOVA followed by the Bonferroni’s test for multiple comparisons for the dose response curve at different times (**a**, **c**). The analysis of the interaction with AM-251 was performed with one-way ANOVA followed by the Bonferroni’s test for the multiple comparison (**b**, **d**). ^∗^*p* < 0.05, ^∗∗∗^*p* < 0.001 vs. vehicle and.^+++^*p* < 0.001 vs. AM-251 + MAM-2201

Visual object response did not change in vehicle-treated mice over the 5 h of observation (Fig. 2a), and the effect was similar to that observed in naïve untreated animals (data not shown). Systemic administration of MAM-2201 (0.01–6 mg/kg; i.p.) significantly and dose-dependently reduced the visual object response in mice [Fig. 2a; significant effect of treatment (F_5,336_ = 376.2, *p* < 0.0001), time (F_7,336_ = 93.61, *p* < 0.0001) and time x treatment interaction (F_35,336_ = 10.97, *p* < 0.0001)]. In particular, the lowest dose (0.01 mg/kg) of MAM-2201 transiently reduced the visual object reflex, at 30 min after injection. Increasing the dose of MAM-2201 (0.1–1 mg/kg) induced long-lasting inhibition at 0.1 mg/kg, while the dose of 1 mg/kg promptly induced a total inhibition of the visual object reflex at 30 min of treatment. The higher doses (3 and 6 mg/kg) tested induced a deep and prolonged impairment of the visual reflexes of mice that lasted until the end of the test, respectively. The inhibition of visual object response induced by MAM-2201 at the highest dose tested (6 mg/kg; (F_3,28_ = 23.93, *p* < 0.0001) was significantly prevented by the pre-treatment with AM 251 (Fig. 2b; 6 mg/kg i.p.; significant effect of treatment (F_3,28_ = 23.93, *p* < 0.0001). MAM-2201 appeared to be slightly more potent than AM-2201 in altering visual responses (Table 2; visual object response test (F_1,76_ = 4.647, *p* = 0.0363)).

**Table 2:** ED_50_ values of AM-2201 and MAM-2201 based on in vivo performed sensorimotor tests. Data are expressed as value ± SEM. ED_50_ (dose of agonist to obtain 50% of the overall mean effect) has been calculated by non-linear regression curve fitting of the dose–response curves determined using the Prism 8.0 software (GraphPad Prism, San Diego CA). ED_50_ curves relative to each test were compared performing the F test. *p < 0.05, **p < 0.01, ***p < 0.001 versus AM-2201. Data relative to AM-2201 elaborated from Bilel et al. (2020)

Sensorimotor test	AM-2201 ED_50_ (mg/kg)	MAM-2201 ED_50_ (mg/kg)
Visual object	1.976 ± 0.063	1.140 ± 0.089^***^
Visual placing	0.500 ± 0.030	0.291 ± 0.025^*^
Startle reflex	1.291 ± 0.010	1.245 ± 0.013
Overall tactile	2.120 ± 0.013	1.861 ± 0.011^**^

### Evaluation of the visual placing response

Visual placing response did not change in vehicle-treated mice during the entire time of the experiment (Fig. 2c). Systemic administration of MAM-2201 (0.01–6 mg/kg; i.p.) significantly and dose-dependently reduced the visual placing response of mice [Fig. 2c; significant effect of treatment (F_5,336_ = 181.8; *p* < 0.0001), time (F_7,336_ = 59.64; *p* < 0.0001) and time x treatment interaction (F_35,336_ = 4.324; *p* < 0.0001)]. In particular, MAM-2201 produced a drastic impairment of visual reflexes and the effect persisted up to 5 h at all the dose tested. The results demonstrate a dose–response relationship with the long-lasting effects induced by the higher doses tested (3 and 6 mg/kg) greater in magnitude than those induced by 0.01–1 mg/kg. The inhibition of visual placing response induced by MAM-2201 at 6 mg/kg was prevented by the pre-treatment with AM 251 6 mg/kg; i.p [Fig. 2d; significant effect of the treatment (F_3,28_ = 30.05, *p* < 0.0001)]. MAM-2201 appeared to be slightly more potent than AM-2201 in altering visual responses in mice (Table 2; visual placing response test (F_1,76_ = 34.73, *p* < 0.0001).

### Evaluation of the acoustic response

**Fig. 3:**
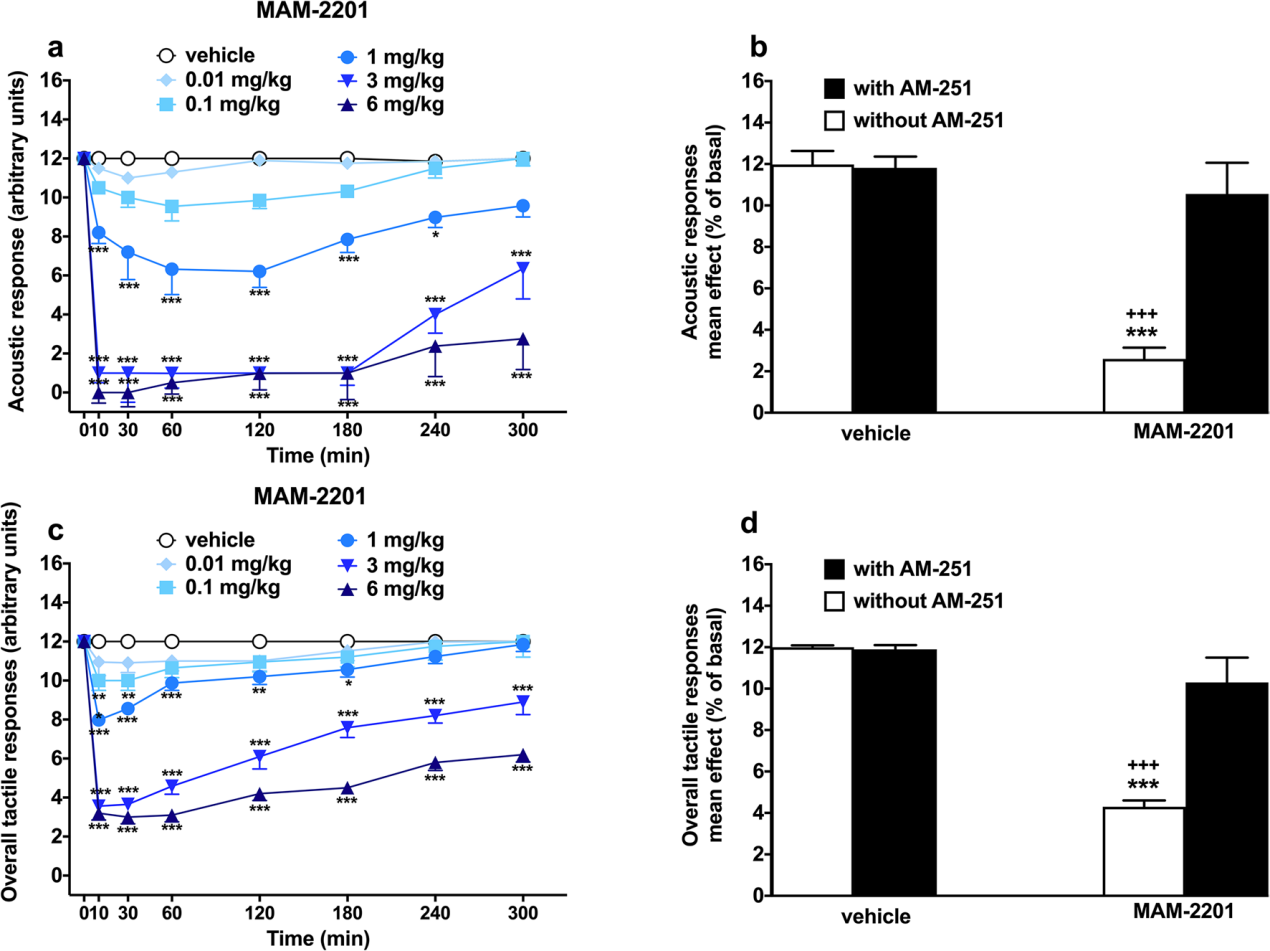
Effect of MAM-2201 (0.01–6 mg/kg i.p.) injections on the acoustic (**a**) and overall tactile (**c**) test in mice. Data are expressed as arbitrary units (**a**–**d**) and represent the mean ± SEM of 8 determinations for each treatment. Interactions of MAM-2201 (6 mg/kg) treatment by pretreatment with the CB1 receptor selective antagonist AM-251 (6 mg/kg, i.p.) are presented in panels **b** and **d**. Statistical analysis was performed by two-way ANOVA followed by the Bonferroni’s test for multiple comparisons for the dose response curve at different times (**a**, **c**). The analysis of the interaction with AM-251 was performed with one-way ANOVA followed by the Bonferroni’s test for the multiple comparison (**b**, **d**). ^∗^*p* < 0.05, **p < 0.01, ^∗∗∗^*p* < 0.001 vs. vehicle and.^+++^*p* < 0.001 vs. AM-251 + MAM-2201

Acoustic response did not change in vehicle-treated mice over the 5-h of observation (Fig. 3a) and the response was similar to that observed in naïve untreated animals (data not shown). Systemic administration of MAM-2201 (0.01–6 mg/kg i.p.) significantly and dose-dependently reduced the acoustic response in mice [Fig. 3a; significant effect of treatment (F_5,336_ = 297.2, *p* < 0.0001), time (F_7,336_ = 40.08, *p* < 0.0001) and time x treatment interaction (F_35,336_ = 7.370, *p* < 0.0001)]. In particular, the acoustic response was immediately reduced to about 20%, 80%, and 100% after MAM-2201 administration at the doses of 1 mg/kg, 3 mg/kg, and 6 mg/kg, respectively. The inhibition of the acoustic response induced by MAM-2201 at 6 mg/kg was prevented by the pre-treatment with AM 251 6 mg/kg i.p. [Fig. 3b; significant effect of treatment (F_3,28_ = 24.57, *p* < 0.0001)]. MAM-2201 appeared to be as potent as AM-2201 in altering acoustic responses (Table 2; acoustic response test).

### Evaluation of the overall tactile reflex

Overall tactile reflex did not change in vehicle-treated mice over the 5 h observation (Fig. 3b) and the response was similar to that observed in naïve untreated animals (data not shown). Systemic administration of MAM-2201 (0.01–6 mg/kg i.p.) significantly and dose-dependently reduced the overall tactile response in mice [significant effect of treatment (F_5,336_ = 404.4, *p* < 0.0001), time (F_7,336_ = 75.80, *p* < 0.0001) and time x treatment interaction (F_35,336_ = 11.94, *p* < 0.0001)]. In particular, MAM-2201 slightly reduced overall tactile response at 1 mg/kg in the first hour after administration. However, a deeper and more persistent effect was induced by higher doses (3 and 6 mg/kg) tested. The inhibition of overall tactile response induced by MAM-2201 at 6 mg/kg was prevented by the pre-treatment with AM 251 6 mg/kg i.p (Fig. 3d; significant effect of treatment (F_3,28_ = 33.48, *p* < 0.0001). MAM-2201 appeared to be more potent than AM-2201 in altering overall tactile responses (Table 2; (F_1,76_ = 9.743, *p* = 0.0025)).

### Evaluationof spontaneous locomotor activity

**Fig. 4:**
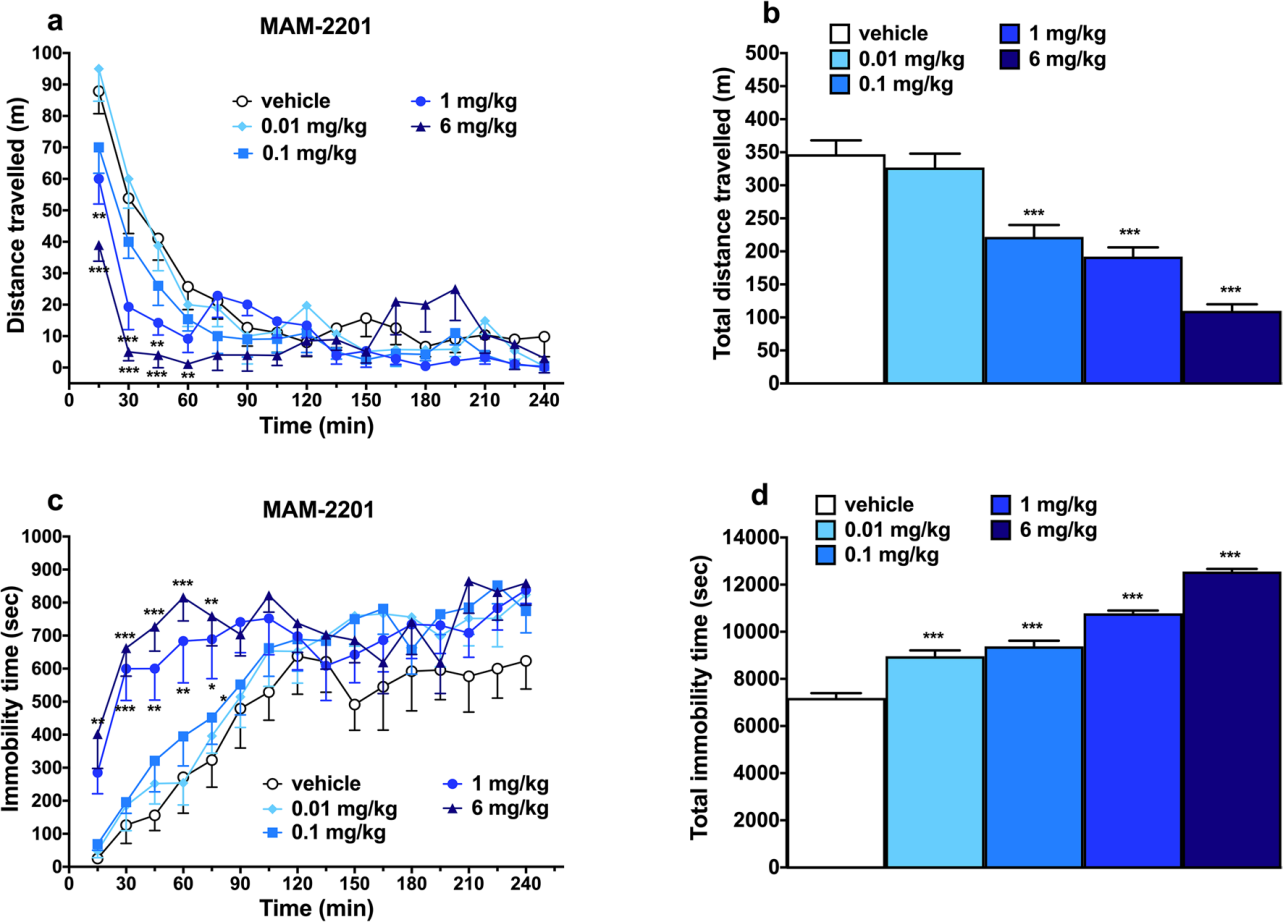
Effect of systemic administration (0.01–6 mg/kg i.p.) of MAM-2201 on the total distance traveled (**a**) and on the total immobility time (**c**) of mice. Overall effect observed in 4-h observation (**b** and **d**). Data are expressed as meters traveled (total distance traveled) and as seconds of immobility (total time immobile) and represent the mean ± SEM of 10 determinations for each treatment. Statistical analysis was performed by two-way ANOVA followed by the Bonferroni’s test for multiple comparisons for the dose response curve of MAM-2201. *p < 0.05, **p < 0.01, ***p < 0.001 versus vehicle

To determine whether the reduction of sensorimotor responses could be due to the inhibition of motor activity, we investigated the effect of MAM-2201 administration (0.01–6 mg/kg; i.p.) on spontaneous locomotor activity in mice. The total distance traveled (Fig. 4a) tended to decrease in vehicle- treated mice during the 5 h observation, while the immobility time (Fig. 4c) increased during the first 120 min and then, remains constant,and these responses are similar to those observed in naïve untreated animals (data not shown). During the first hour of the experiment, MAM-2201 at 1 and 6 mg/kg reduced the total distance traveled [Fig. 4a; significant effect of treatment (F_4,720_ = 12.95, *p* < 0.0001), time (F_15,720_ = 44.51, *p* < 0.0001) and time x treatment interaction (F_60,720_ = 3.001, *p* < 0.0001)] and increased the immobility time [Fig. 4c; significant effect of treatment (F_4,720_ = 24.32, *p* < 0.0001), time (F_15,720_ = 19.38, *p* < 0.0001) and time x treatment interaction (F_60,720_ = 1.493, *p* = 0.0112)] in mice. The comparison of total average effects induced by the MAM-2201 revealed a significant effect of treatment on both the total distance traveled (Fig. 4b; F_4,45_ = 32.08, *p* < 0.0001) and the immobility time (Fig. 4d; F_4,45_ = 109.1, *p* < 0.0001).

### Prepulse inhibition study

**Fig. 5:**
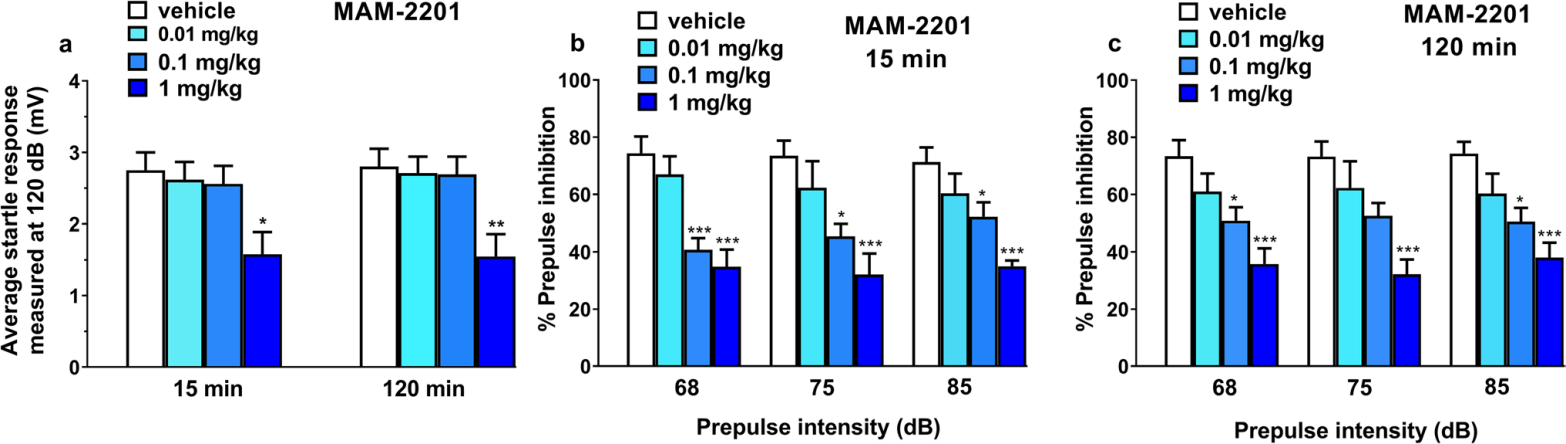
Effect of MAM-2201 (0.01–1 mg/kg; i.p.) on startle amplitude (**a**) and pre-pulse inhibition (PPI; **b**, **c**) in mice. Effects on PPI are shown for the three pre-pulse intensities (68, 75, and 85 dB), 15 and 120 min after treatment (**b**, **c**). Data are expressed (**a**, **b,** and **c**) as mean ± SEM of 10 animals for each treatment. The statistical analysis was performed with a one-way ANOVA followed by Bonferroni’s test for multiple comparisons. *p < 0.05, **p < 0.01, ***p < 0.001 versus vehicle

Vehicle injection did not change startle and PPI response in mice (Fig. 5a–c) and the effect was similar in naïve untreated animals (data not shown). Systemic administration (i.p.) of MAM-2201 (0.01–1 mg/kg;i.p) reduced startle amplitude in mice only at 1 mg/kg (Fig. 5a), both at 15 (treatment F_3,36_ = 4.067, *p* = 0.0138) and 120 min treatment (F_3,36_ = 5.114, *p* = 0.0046). In addition, MAM-2201 at 0.1 and 1 mg/kg inhibited the PPI in mice after 15 min of its administration (Fig. 5b). In addition, ANOVA analysis detected a significant decrease of prepulse startle intensity with 68 dB respectively; treatment (F_3,36_ = 11.38, *p* < 0.0001), 75 dB (~ 28% and ~ 41%, respectively, treatment (F_3,36_ = 7.169, *p* = 0.0007) and 85 dB (~ 19% and ~ 37%, respectively;treatment F_3,36_ = 9.000, *p* = 0.0001). One-way ANOVA also detected a significant effect of treatment 120 min from MAM-2201 administration (Fig. 5c) at prepulse intensities of 68 dB (~ 19 and 40% for dosages of 0.1 and 1 mg/kg (F_3,36_ = 11.38, *p* < 0.0001)), 75 dB (~ 34% at the dose of 1 mg/kg (F_3,36_ = 7.169, *p* = 0.0007)) and 85 dB (~ 19% and ~ 37% at dosages of 0.1 and 1 mg/kg (F_3,36_ = 9.000, *p* = 0.0001)).

### Novel Object Recognition test MAM-2201 and AM-2201

**Fig. 6:**
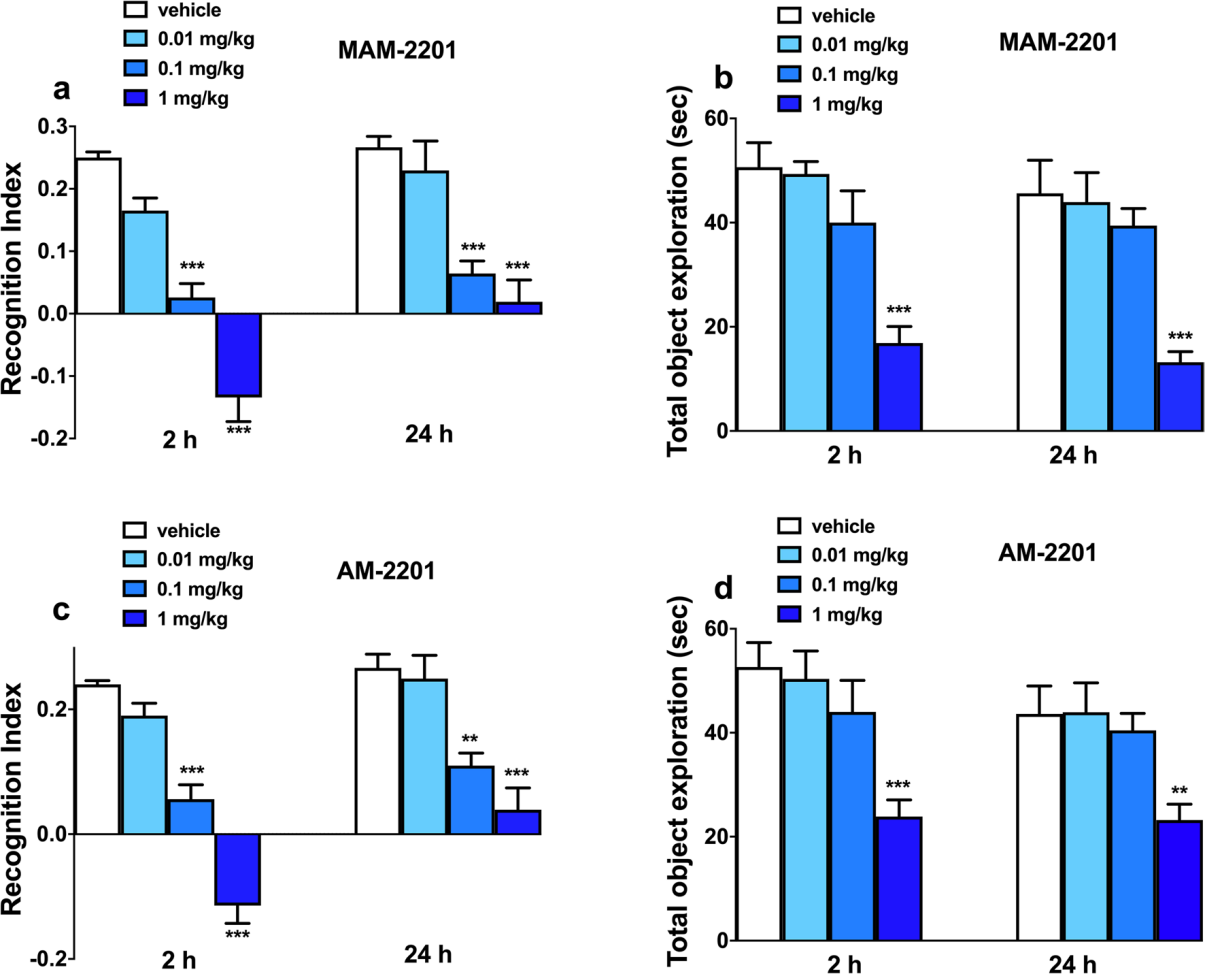
Effect of systemic administration (0.01–1 mg/kg i.p.) of MAM-2201 (**a**) and AM-2201 (**c**) on recognition index (RI) in the novel object recognition (NOR) test in mice. Both test compounds given 15 min after the familiarization phase impaired the short- (at 2 h) and long-term (24 h) object memory recognition in mice. Data are expressed as RI (see material and methods) and represent the mean ± SEM of 10 animals for each treatment. Statistical analysis was performed by one-way ANOVA followed by the Bonferroni’s test. ***p < 0.001, **p < 0.01 versus vehicle. Effect of systemic administration (0.01–1 mg/kg i.p.) of MAM-2201 (**b**) and AM-2201 (**d**) on total object exploration (TOE) in the NOR test in mice. Compounds given 15 min after the familiarization phase impaired the TOE both at 2 and 24 h. Data are expressed as absolute values (s) and represent the mean ± SEM of 10 animals for each treatment. Statistical analysis was performed by one-way ANOVA followed by the Bonferroni’s test. ***p < 0.001, **p < 0.01 versus vehicle

To investigate whether novel synthetic cannabinoid agonist MAM-2201 and AM-2201 affect memory retention in mice, we performed the NOR test (Fig. 6a–d). During the familiarization phase, no difference was seen in the time spent by mice to investigate the two objects (data not shown). There were no significant differences between vehicle-treated and control mice in the NOR test (2 h after vehicle injection: *t* = 0.2456, df = 18, *p* = 0.8088; and 24 h: *t* = 0.1438 df = 18, *p* = 0.8873; data not shown).

NOR was impaired both at 2 and 24 h from the administration of MAM-2201 (Fig. 6a; F_3,36_ = 45.99; *p* < 0.0001 and F_3,36_ = 14.17; *p* < 0.0001, respectively) and AM-2201 (Fig. 6c; F_3,36_ = 56.13; *p* < 0.0001 and F_3,36_ = 13.88; *p* < 0.0001, respectively). Specifically, MAM-2201 at 0.1 mg/kg significantly reduced the RI at 2 h, while it produced a negative score (indicating a mouse preference toward the familiar object (A) with respect to the novel one (B)) following the administration of 1 mg/kg. Moreover, the effect of MAM-2201 persisted at 24 h, with a significant decrease observed at both 0.1 and 1 mg/kg. Similarly, 2 h after AM-2201 administration, the results indicate a RI reduction (0.1 mg/kg) or reversal (1 mg/kg). The AM-2201 induced memory impairment persisted at 24 h as indicated by the significant reduction of RI observed at the 0.1 and 1 mg/kg doses.

The Total Object Exploration (TOE) time was then calculated to d the effects of cannabinoids administration on the mice ability of the mice to explore the objects in the NOR test. There were no differences in TOE time between the untreated control animals and vehicle-treated mice (2 h after the vehicle administration: *t* = 0.2493, df = 18, *p* = 0.8059; 24 h: *t* = 0.5098, df = 18, *p* = 0.6164; data not shown). However, the TOE time was impaired at 2 and 24 h after both MAM-2201 (Fig. 6b; F_3,36_ = 13.08; *p* < 0.0001 and F_3,36_ = 10.49; *p* < 0.0001, respectively) and AM-2201 (Fig. 6d; F_3,36_ = 6.990; *p* = 0.0008 and F_3,36_ = 4.825, *p* = 0.0063, respectively;) administration at 1 mg/kg dose.

## Discussion

### Affinity and potency of the examined compounds

MAM-2201 and AM-2201 retain nanomolar affinity for both murine and human CB_1_ and CB_2_ receptors with a preference for CB_1_ receptor (Table 1). As previously demonstrated for other synthetic cannabinoids (Vigolo et al. 2015), the high affinity was associated with MAM-2201 and AM-2201 potency values in inhibiting cyclic AMP formation. From a structural point of view, MAM-2201 is a naphtoyl-indole derivative (Hess et al. 2015; Uchiyama et al. 2013), which differs from its analogue AM-2201 by the presence of a methyl substituent on carbon 4 (C-4) of the naphthoyl moiety (Fig. 1). Particularly, it has been ascertained that both electron withdrawing and electron donating substituents (Wiley et al. 2012, 2017) in this position do not adversely affect the aromatic stacking that is considered to be of considerable importance for cannabinoid receptor binding affinity (Reggio et al. 1998; Huffman et al. 2003).

### Sensorimotor and motor responses

MAM-2201 altered in vivo sensorimotor responses in mice with similar potency values (Table 2) respect to AM-2201, appearing to be slightly more potent in altering visual and tactile responses. However, it should be noted that data about effects of MAM-2201 and its analogue AM-2201 (Bilel et al. 2020) have been collected in different experimental sessions. Acute systemic administration of MAM-2201 provoked a profound sensorimotor impairment, leading to nearly total inhibition of sensorimotor reflexes at the higher doses (1–6 mg/kg) tested. Our results are consistent with previously reported detrimental effects of AM-2201, halogenated JWH-018 derivatives (Bilel et al. 2020) and other JWH-type SCs (Ossato et al. 2015, 2016). These effects were fully prevented by pre-treatment with the CB_1_ receptor selective antagonist/inverse agonist AM-251. Thus, these data support the hypothesis that the sensorimotor impairments induced by MAM-2201 up to 6 mg/kg are fully dependent on CB_1_ receptor stimulation (Vigolo et al. 2015; Ossato et al. 2015, 2016; Bilel et al. 2020). Accordingly, these substances would bind to and activate CB_1_ receptors located presynaptically in circuitries designated for sensorimotor responsiveness and impair their normal functioning (Gomez-Nieto et al. 2014; Reig and Silberberg. 2014; Yoneda et al. 2013; Dasilva et al. 2012; Hemelt and Keller. 2008; Tzounopoulos et al. 2007; Price et al. 2003). In fact, a subpopulation of neurons selectively localized in the dorsomedial striatum of mice has been previously linked to processing of visual information (Reig and Silberberg 2014). Similarly, the acoustic startle reflex is induced by the activation of three serially connected neuronal pathways that involve the dorsal cochlear nucleus (Gomez-Nieto et al. 2014). Furthermore, suppression of central trigeminal nerve transmission has been observed after administration of SCs (Jenkins et al. 2004; Papanastassiou et al. 2004), as well as reduced cornea-evoked trigeminal brainstem activity (Bereiter et al. 2002).

The spontaneous locomotor activity assay further demonstrated the profound motor deficits induced by MAM-2201, which were observed as an increase in immobility time accompanied by a corresponding decrease in the distance traveled in the open field test. MAM-2201 caused a disruption of motor activity starting from the lowest doses (0.01–0.1 mg/kg) tested, consistent with the effects previously reported for its analogue AM-2201 (Bilel et al. 2020). Therefore, these data further illustrate the detrimental effects of cannabinoid agonists on locomotor activity and catalepsy that are typically reported as “tetrad effects” induced by cannabinoids in rodents (Ossato et al. 2016; Vigolo et al. 2015). Notably, it has been shown that THC (De Giacomo et al. 2020) and SCs may regulate motor activity by acting on CB1 receptors located in the cerebellum and basal ganglia (Funada et al. 2020; Morera-Herreras et al. 2012; Rodriguez de Fonseca et al. 1998). Specifically, they could affect motor tasks by altering dopaminergic motor circuits or central glutamate neurotransmission (Funada et al. 2020; Morera-Herreras et al. 2012).

### Cognitive functions

MAM-2201 administration induced a dose-dependent alteration of the startle reflex accompanied by a reduction of the prepulse inhibition and impaired short- (2 h) and long-term (24 h) working memory in mice, also observed after AM-2201 administration. These results agree with our previous studies, which showed for the first time impaired prepulse inhibition (Bilel et al. 2020) and disruption of memory functions (Barbieri et al. 2016) after exposure to JWH-018 and its halogenated derivatives. Moreover, cannabinoids can elicit a disruption of the prepulse inhibition in both humans (Kedzior and Martin-Iverson 2006) and animals (Peres et al. 2016), which has been associated with cannabis-induced psychosis (Morales-Muñoz et al. 2017) and also schizophrenic syndromes (Javitt and Zukin 1991). Further studies showed the coexistence of PPI and cognitive deficits in diseases such as schizophrenia (Geyer 2006), suggesting a potential link between prepulse inhibition and working memory performance in mice (Singer et al. 2013). In this context, the potential role of CB_1_ receptors in modulating the release of neurotransmitters (dopamine, serotonin and glutamate; Howlett et al. (2004)) implicated in schizophrenia and psychosis have been shown (Sawa and Snyder 2003), thus suggesting that SCs can indirectly modulate a variety of psychosis-related receptors (D_2_, 5-HT_2A_ and NMDA; Fantegrossi et al. 2018). Indeed, CB_1_-mediated impaired auditory gating and abnormal neuronal synchrony have been reported after the administration of CP-55940 in rats (Hajós et al. 2008). It is worth noting that also the effect of MAM-2201 and other SCs on memory functions could be at least partly mediated by CB_1_ receptors highly expressed in the hippocampus and perirhinal cortex (Basavarajappa and Subbanna 2014; Marsicano and Lafenêtre 2009; Marsicano and Lutz 1999) involved in normal memory functions (Baxter 2010). In particular, it has been highlighted that intraperirhinal cortex administration of the synthetic cannabinoid HU-210 alters object recognition memory in rats (Sticht et al. 2015), and intracerebroventricular infusion of 5F-AMB disrupts acquisition of recognition memory and the effect is prevented by the coinfusion of AM-251 (Ito et al. 2019).

RI changes in the NOR test have been observed 2 and 24 h after MAM-2201 and AM-2201 administrations (0.1–1 mg/kg). These effects are not due to a reduction in locomotor activity, since the tested compound affected motor capacity of mice only during the first 60 min in the open field test. The same can be assumed for AM-2201, given our previous study reporting that it reduced the distance traveled by mice up to 45 min (Bilel et al. 2020). On the other hand, the disruption of the working memory was still observed in the NOR test performed 24 h after its administration. It is worth noting that a low dosage of MAM-2201 and AM-2201 (0.1 mg/kg) did not alter the TOE time, while the highest dose (1 mg/kg) administered induced a reduction of this parameter. However, severe sensory gating deficits were also observed 120 min after the injection of 1 mg/kg of MAM-2201 (Fig. 5). Thus, these findings confirm that abuse of these SC substances might result in severe information processing and sensory impairments as demonstrated by the numerous intoxication cases reported in the literature over the last years (Yeruva et al. 2019; Fattore 2016; Hermanns-Clausen et al. 2013; Every-Palmer 2010, 2011).

Administration of the highest dose of MAM-2201 and AM-2201 caused a greater exploration of the familiar object relative to the novel object (RI reversal). It is important to note that the test compounds were administered at a sufficient time (15 min) to acquire memory of the objects (A, A) during the familiarization phase. Therefore, this could be linked to a drug-induced impairment in the already acquired memory (Ennaceur 2010). In our experimental conditions, it cannot be excluded that this effect is due to alterations in sensory functions induced by these compounds (Bilel et al. 2020), and further studies are required to address this issue. As previously reported with other JWH-018 analogs, the acute administration of MAM-2201 and AM-2201 impairs NOR in mice causing RI changes detectable at 2 and 24 h after their administration. The long-lasting cognitive deficits observed in the NOR could also be linked to the pharmacokinetic features of these compounds. Specifically, Cannaert and colleagues have shown metabolites of both methylated (MAM-2201) and ethylated (EAM-2201) analogs of AM-2201 exhibiting agonist activity at both CB_1_ and CB_2_ receptors (Cannaert et al. 2016). Further studies of correlation between pharmacological and pharmacokinetics of MAM-2201 and AM-2201 and their metabolites should be performed to confirm this hypothesis.

### Implications in forensic medicine

**Fig. 7:**
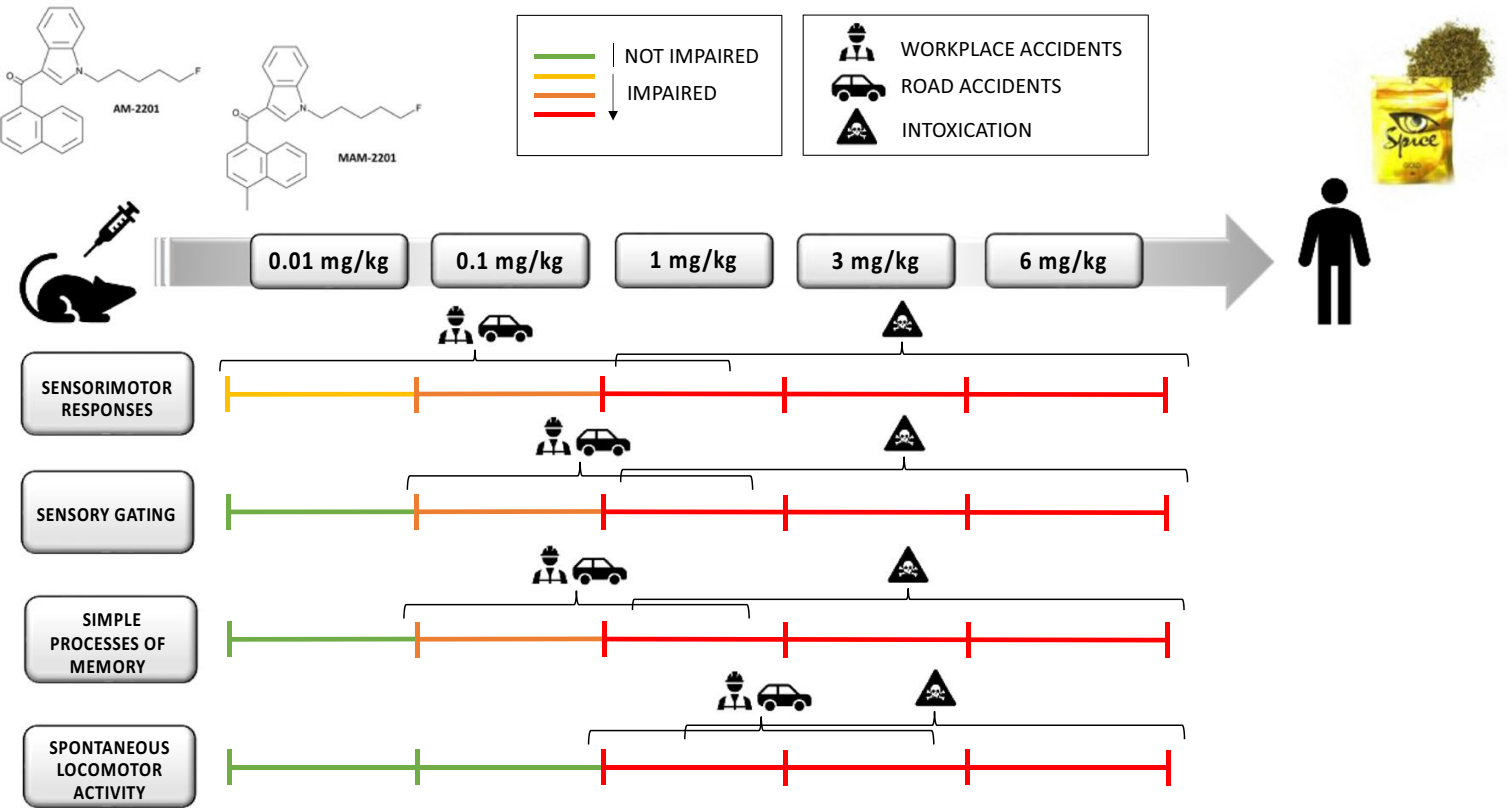
Effects induced by systemic administration of MAM-2201 (sensorimotor responses, spontaneous locomotor activity and sensory gating) or MAM-2201 and AM-2201 (working memory) in mice and possible forensic implications

As suggested by the presence of SCs in biological samples of drivers involved in cases of Driving Under the Influence of Drugs (DUID; Musshoff et al. 2014; Tuv et al. 2014), the use of SCs clearly leads to serious impairments that are not compatible with safe driving (Theunissen et al. 2021). As with drivers (Orazietti et al. 2022), exposure to these psychoactive substances may lead to an increased risk of suffering work-related severe injuries or death for those who are involved in hazardous working activities (Howard and Osborne 2020; Musiał et al. 2022). In fact, the risk concern regards also personnel that may encounter NPS as part of their work in a range of operational settings, which may result in increased health risk from occupational exposure (Dobaja et al. 2017; Tapp et al. 2014; EMCDDA 2022a, b). Another issue involves the consumption of NPS in the workplace during working hours, facilitated by the fact that SCs are not always identified on random work drug screens. The increasing use of SCs and other NPS may be associated with an increase in the frequency and severity of labor accidents, as well as the workers’ poor general state of health and productivity, generating higher costs for enterprises (Frone 2013; Dinis-Oliveira and Magalhaes 2020). The present study has demonstrated that MAM-2201 produces profound deficits in sensory information processing, sensory gating, and working memory in mice, emphasizing the health risks related to MAM-2201 consumption (Fig. 7). It is worth noting that sensorimotor and cognitive impairment have been shown to appear at dosages that do not alter motor capacity of mice. In line with this evidence, previous studies have also pointed out that both altered sensorimotor (Ossato et al. 2015) and electrophysiological functions (Barbieri et al. 2022) can be observed in SCs-treated mice at dosages and time points that have not been related to motor impairment. Thus, such in vivo alterations confirm that SCs consumption could contribute to the severe general impairment and suggest the urgent need of considering additional surveillance research for detecting the use of NPS among drivers and workers.

## Data Availability

The authors declare that the data supporting the findings of this study are available within the paper and its Supplementary Information files. Should any raw data files be needed in another format they are available from the corresponding author upon reasonable request. Source data are provided with this paper.

## Abbreviations

NPS: Novel psychoactive substances
JWH-018: 1-Pentyl-3-(1-naphthoyl) indole
JWH-018-R: AM-2201(1-(5-fluoro-pentyl)-3-(1-naphthoyl) indole)
MAM-2201: (1-(5-Fluoropentyl)-1H-indol-3-yl)(4-methylnaphthalen-1-yl) methanone
AM-251: 1-(2,4-Dichlorophenyl)-5-(4-iodophenyl)-4-methyl-N-(piperidin-1-yl)-1H-pyrazole-3-carboxamide
THC: Tetrahydrocannabinol
